# Understanding CEAP Classification: Insights from an Italian Survey on Corona Phlebectatica and Recurrent Active Venous Ulcers by Vascular Specialists

**DOI:** 10.3390/medicina60040618

**Published:** 2024-04-10

**Authors:** Gianfranco Lessiani, Luca Gazzabin, Giulio Cocco, Antonio Corvino, Damiano D’Ardes, Andrea Boccatonda

**Affiliations:** 1 Angiology Unit, Internal Medicine Department, Villa Serena Hospital, 65013 Città Sant’Angelo, Italy; 2 Vascular Ulcers and Diabetic Foot Surgery Unit, Donatello Private Hospital, 50019 Firenze, Italy; 3 Department of Neuroscience, Imaging and Clinical Sciences, “G. d’Annunzio” University, 66013 Chieti, Italy; 4 Medical, Movement and Wellbeing Sciences Department, University of Naples “Parthenope”, 80133 Naples, Italy; 5 Department of Medicine and Aging Science, Institute of “Clinica Medica”, “G. d’Annunzio” University of Chieti, 66100 Chieti, Italy; 6 Department of Medical and Surgical Sciences, University of Bologna, 40138 Bologna, Italy

**Keywords:** venous chronic disease, CEAP classification, corona phlebectatica, venous ulcers

## Abstract

*Background and Objectives:* The clinical relevance of “corona phlebectatica” and the management of risk factors for recurrence of venous ulcers in patients with chronic venous disease may be variable based on vascular specialists in different geographical areas of Italy. The aim of the present survey is to evaluate the management of patients with chronic venous disease by vascular specialists in different areas of the national territory. In particular, this involves ascertaining the clinical/prognostic relevance attributed to the presence of the “corona phlebectatica” as well as to the management of risk factors related to recurrence of venous ulcers. *Materials and Methods:* The web-based survey aimed at vascular medicine specialists with particular interest in venous disease. A questionnaire was developed, based on 12 questions, in relation to clinical assessment, risk factor management, and therapy in patients with chronic venous disease. *Results:* Almost all of the specialists involved actively participated in the survey, declaring that they personally manage chronic venous disease overall. There was a strong agreement in the prognostic consideration attributed to the presence of “corona phlebectatica” and to the management of risk factors for venous ulcer recurrence, regardless of the different geographical areas of interest. *Conclusions:* Accordingly with the results of this self-assessment survey, the skills and experience of the specialists involved appear to be of a good standard, both in the clinical evaluation and in the management of the progression of chronic venous disease. However, the need to reach more cultural insights into the correlations between chronic venous disease and risk factors correlated with disease progression emerges. Moreover, there was the need for a greater and tighter overall clinical control of a patient with chronic venous disease, also in relation to the presence of comorbidities.

## 1. Introduction

In past years, the term “chronic venous disorders” has been used to include the overall spectrum of functional or morphological alterations of the venous system in the general definition of venous pathology. Otherwise, the term “chronic venous insufficiency (CVI)” is used for the advanced forms, in which the alterations of the venous system induce oedema, skin changes, venous ulcers [1].

CVI can be caused by primary varicosis or a post-thrombotic syndrome. Both lead to venous hypertension, which in turn leads to microvascular changes such as the elongation of capillaries, micro-thrombosis, perivascular fibrin around vessels, and leakage of leukocytes [2].

The diagnosis of CVI is based on clinical characteristics; there are skin changes that are caused by chronic venous hypertension: oedema, capillaries visible around the ankle, changes in trophic skin such as hyperpigmentation caused by deposits of hemosiderin, white atrophy, hardening of the underlying skin and tissues (dermatoliposclerosis), and stasis eczema.

The CEAP classification (Clinical, Aetiological, Anatomical, Pathophysiological) is the most widely used system to classify chronic venous disease (CVD). The CEAP is a descriptive system that allows us to identify the state of the venous pathology at a specific time point [3,4].

The CEAP classification was revised in 2020 with reference to new sub-categories concerning “corona phlebectatica” (C4c), varicose veins, and recurrent venous ulcers (C2r; C6r). Moreover, the subdivision of etiological causes between intravenous (Esi) and extravenous (Ese) and the new abbreviations for the anatomical description were included [5].

**Figure 1:**
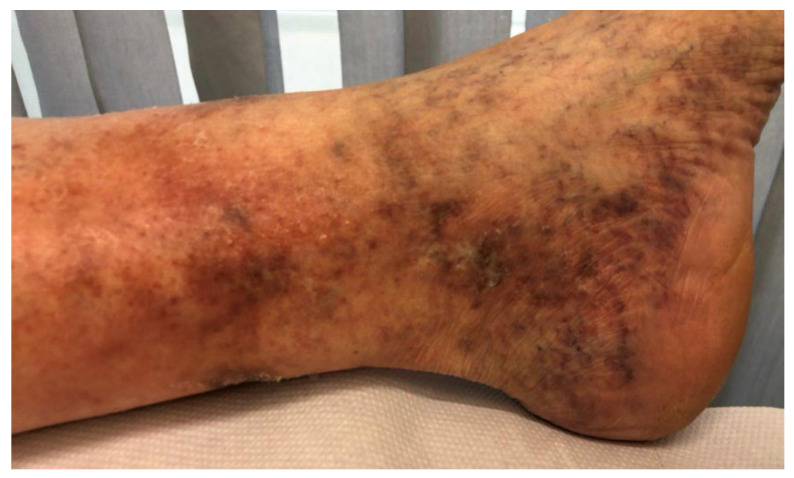
Example of corona phlebectatica.

The definition of corona phlebectatica is based on the abnormal visibility of cutaneous blood vessels around the ankle, consisting of four distinct elements: “venous cups”, telangiectases in blue and red, and capillary regions named as “stasis spots” [6,7] (Figure 1).

Stasis spots and blue telangiectases were directly related to the ascending order of “C” classes in CEAP classification [7]. Perforant veins of the medial and lateral surfaces of the foot constitute the anatomical ground for the formation of the corona phlebectatica and are component parts of the neurovascular bundle [8]. From four to six perforant veins can be found on the medial surface of the foot [8]. They directly connect the medial marginal vein and vv. plantaris medialis. From two to three perforant veins are found on the lateral surface of the foot. They directly connect the lateral marginal vein and vv. plantaris lateralis [8]. Corona phlebectatica has been considered a risk factor for chronic skin ulceration together with the increased severity of CVD and BMI, prior history of DVT, smoking, and lipodermatosclerosis [9].

CVD is a very common disease. Epidemiological data from a recent systematic review show an extremely significant prevalence of the various CEAP classes—C0: 9%; C1: 26%; C2: 19%; C3: 8%; C4: 4%; C5: 1%; and C6: 0.6% [10]. The annual incidence of class C2 ranges between 0.2% and 2.3%; CVD progresses in 31.9% of patients over a 13.4-year time period [11].

Notably, patients in the CEAP-C2 class display a rate of progression towards the development of venous leg ulcers (VLUs) of 22% in 6 years [11].

VLU affects 1% of the population with a prevalence that increases with age [12]. In the United States, up to 4% of people over the age of 65 are suffering from it [12]. VLUs are usually located on the medial side of the leg and the medial ankle. However, a minority is determined by an isolated varicosis of the small saphenous vein or a congenital aplasia of the venous valves and is found, respectively, on the lateral or dorsal part of the foot [13].

Regarding CVD progression, the Edinburgh vein study showed that 0.9% of the population develop venous reflux annually [14]. Another study showed that CVD progression occurs in approximately 58% of patients over a 13-year time period, with an annual rate of 4.3%. Furthermore, another study showed that approximately 30% of patients with uncomplicated varicose veins at baseline presented skin changes over time [15].

According to the most recent guidelines on CVD, female gender, pregnancy, obesity, age, prolonged standing, and positive family history are the most common risk factors [11]. The most severe form of CVD is characterized by the presence of VLUs, and it represents a particularly serious condition for the patient and is extremely impactful also from a social and economic point of view. The prevalence of VLU is about 1% in the general population, but it can reach about 3% in the population over 80 years of age [16]. Some literature sources showed that about 7% of ulcers persist over a 5-year period [17]. Some studies showed that the recurrence rate of healed VLUs is approximately 70% at three months [18,19].

According to the new CEAP classification, an attempt was made to better understand the current management of patients with “corona phlebectatica” (C4c) and with relapsing venous ulcers (C6r) in clinical practice, administering a survey to vascular specialists, who deal with their day-to-day management of CVD.

Moreover, lymphatic involvement was demonstrated in mild and moderate (C2–C4) venous insufficiency [20]; in particular, the degradation of lymphatic anatomy and function was shown with the progression of CVD as compared to healthy subjects [20]. Abnormal lymphatic architecture, including interstitial backflow, dermal backflow, vessel segmentation, dilation, and/or unusual drainage patterns, was observed in all CVD limbs, with severity generally progressing with venous insufficiency classification [20].

Some authors suggested that by evaluating near-infrared fluorescent lymphatic imaging, lymphatic varicosities, and resulting lymphatic insufficiency, can accompany the development of venous varicosities in C2 disease and are especially prevalent in C3 disease, where limb swelling is the distinguishing clinical feature [20].

The aim of this research is to describe what vascular specialists practicing in Italy and managing people with venous disease know about “corona phlebectatica” and relapsing venous ulcers and their practices to assess, manage, and prevent them.

## 2. Material and Methods

A web survey was performed about the evaluation of the CEAP classification, and in particular on the presence of “corona phlebectatica” and on the prevention of venous ulcers by vascular specialists (angiologists, vascular surgeons, phlebologists). The survey evaluated behaviours and types of therapy used by vascular specialists in clinical practice in the management of CVD risk factors and to counter its progression.

The estimated sample size was 72 participants, by considering that there were a total of 87 physicians in the VIVA group, and by calculating a margin of error of 5% and a 95% confidence interval (with response distribution of 50%).

A questionnaire was created, based on 12 questions, collected in three main groups:

- Clinical area: Clinical evaluation in relation to the presence of “corona phlebectatica” and its clinical/prognostic relevance, as well as the frequency and relevance of venous ulcer recurrences in patients with CVD.
- Risk factors: Considerations and behaviours of specialists in relation to risk factors for the evolution of CVD.
- Therapies: Considerations and therapeutic behaviours (physical, pharmacological, and surgical) in the management of a patient with CVD. In particular, they considered the presence of “corona phlebectatica” and the prevention of recurrence of venous ulcers.

The first draft of the questionnaire underwent revisions and retesting by the board members of the VIVA (View on Vascular Disease) working group (VIVA, composed of 3 vascular medicine specialists and 3 vascular surgeons). The VIVA working group executive committee approved the final questionnaire. The ethical approval was waived by the ethics committee due to the nature of this study. This study was conducted in accordance with the Declaration of Helsinki (as revised in 2013). The survey was composed of multiple-choice questions.

The REDCap online survey was distributed from June to October 2020. All responses were voluntary and anonymous, and electronic informed consent was obtained from responders.

In June 2020, the questionnaire was sent to 87 vascular specialists. Eligibility criteria to join the survey were to be a vascular specialist and to carry out clinical activity in the vascular field, particularly in the diagnosis and management of CVD. Some questions in the questionnaire were multiple choice. The questionnaire was completed by the participants in the period from June to October 2020 and was sent with a “cover letter” explaining the rationale and methods of carrying out the survey. The data collected were analysed with descriptive statistics methods returning frequency, percentage, and mean values.

## 3. Results

**Figure 2:**
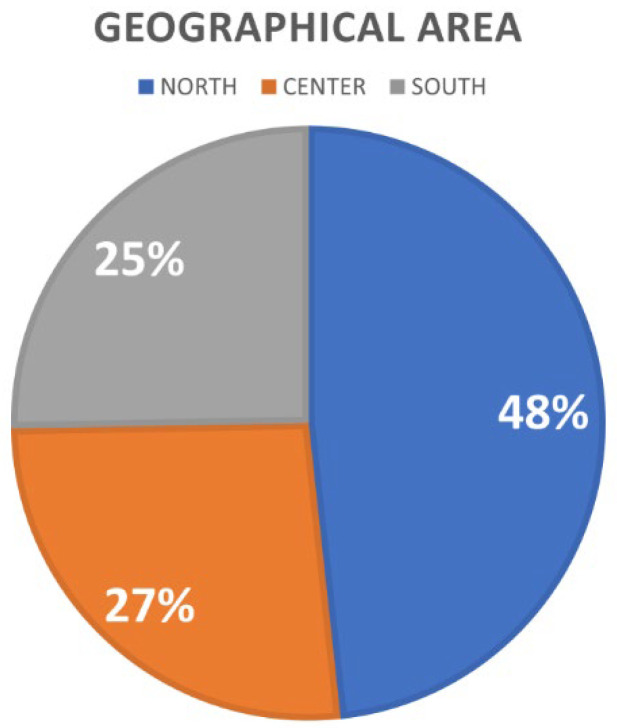
Italian geographical area to which the survey respondents belong.

**Figure 3:**
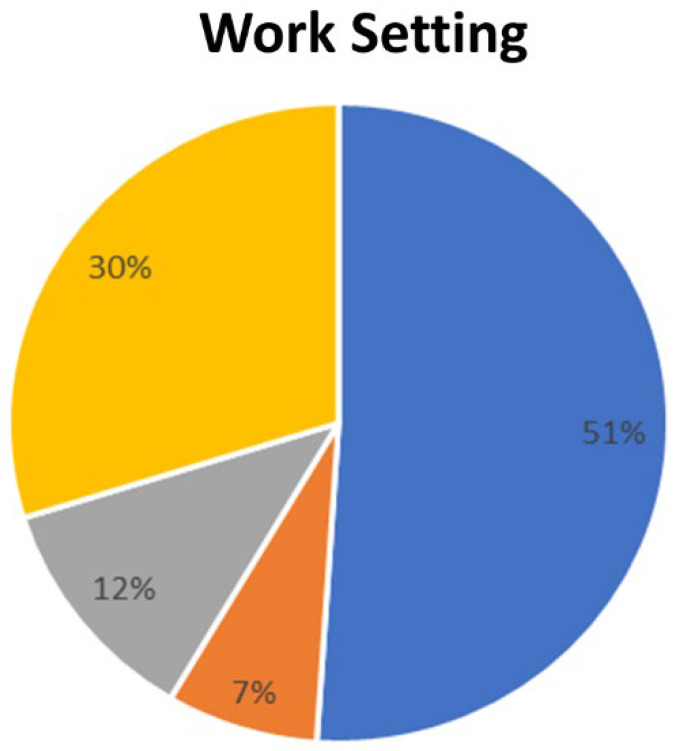
Work setting of survey respondents; yellow: physicians working as freelancers; orange: physicians working in private hospitals; grey: physicians working in private hospitals affiliated with the national healthcare service; blue: physicians working in public hospitals.

**Figure 4:**
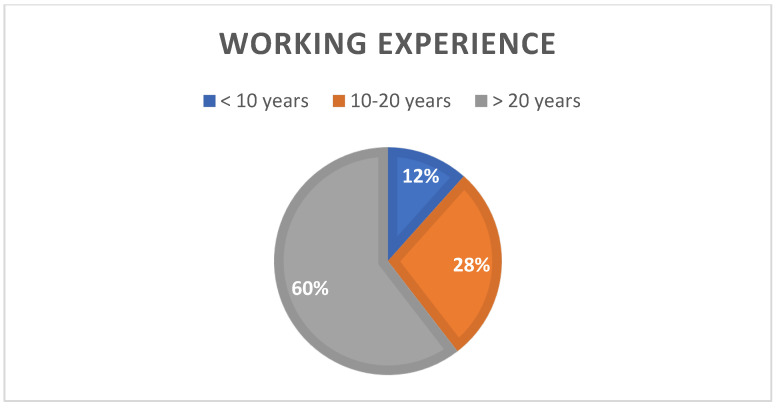
Working experience of survey respondents.

**Figure 5:**
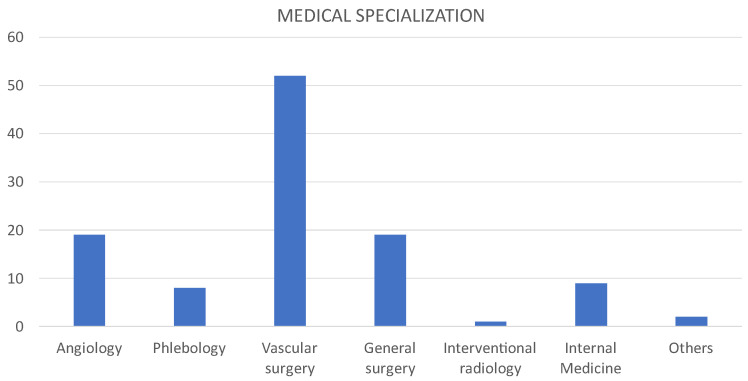
Medical specialization of survey respondents.

All 87 vascular specialists responded to the questionnaires, which were all useful for the statistical analysis. Figure 2, Figure 3, Figure 4 and Figure 5 show the information relating to the survey participants in relation to work structure, region of employment, years of activity, and specialization. Figure 2 shows a greater representation of specialists in the macro-geographic area of northern Italy (55.2%).

Most of the respondents were employed in hospitals (55.2%), and 32.2% worked as a private physician. All specialists demonstrated extensive professional experience. Indeed, almost 60% of them had professional experience of 20 years or more. The majority of respondents were specialists in vascular surgery, 21.8% were angiologists and general surgeons, and 9.25 were phlebologists.

### 3.1. Clinical Area

Almost 50% (49.4%) found the presence of “corona phlebectatica” among between 20 and 50% of their patients. About 73.6% considered the presence of “corona phlebectatica” a moderate risk for the development of VLUs. On the other hand, 23.0% consider it a “high” risk factor. In the clinical experience of 55.2% of respondents, the risk of recurrence of venous ulcers is over 15%, while it settles between 5 and 10% for 41.4% of respondents. About 96.6% of specialists considered the possibility of a venous ulcer recurrence within 1–5 years. No respondent considered the possibility of recurrence beyond 5 years.

### 3.2. Risk Factors

**Figure 6:**
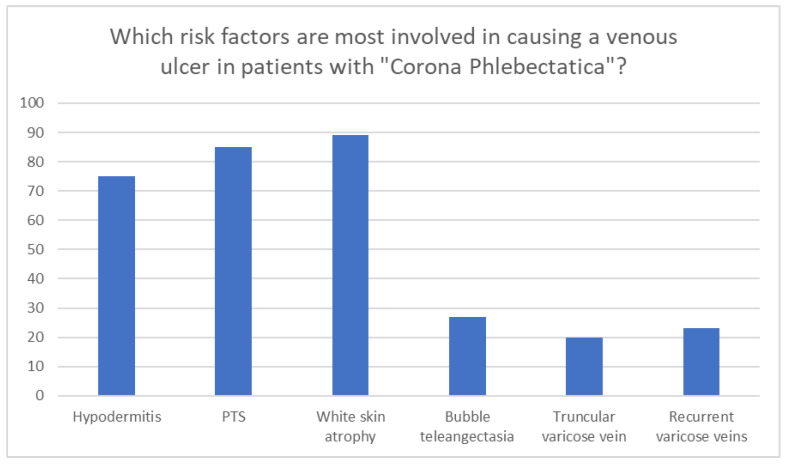
Risk factors for venous ulcer in patients with corona phlebectatica. Post-thrombotic syndrome, PTS.

White atrophy (89.7%), post-thrombotic syndrome (PTS) (85.5%), and hypodermitis (75.9%) were the most considered risk factors for the onset of venous ulcers by respondents (Figure 6). About 80.5% argued that the presence of a PTS represents a high risk for a recurrence of a venous ulcer. The main risk factors for ulcer recurrence were poor patient compliance with therapy (96.6%), non-use of compression therapy (94.3%), obesity (87.4%), not undertaking medical therapy (75.9%).

### 3.3. Therapy

**Figure 7:**
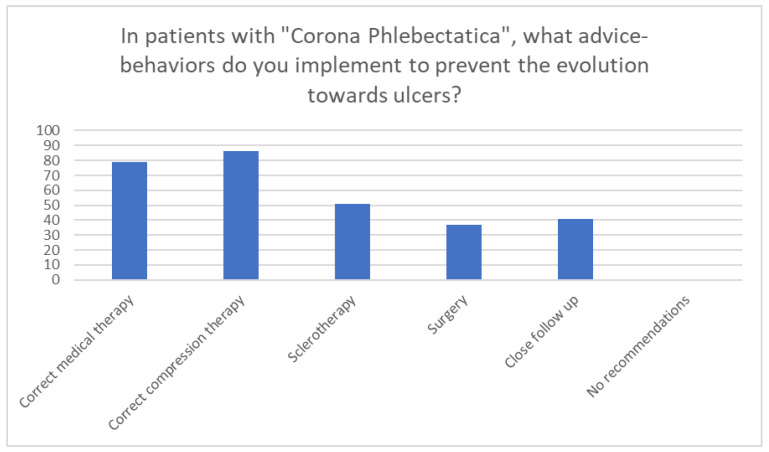
The summary scheme on the behaviours/therapeutic options to be recommended to prevent the evolution towards ulcers.

In patients with corona phlebectatica, almost all specialists considered it possible to contrast/delay the appearance of VLUs (97.7%). (1) In patients with corona phlebectatica, participants suggested the sequent advice–behaviours to prevent the evolution towards ulcers: correct medical therapy (90.8%), correct compression therapy (98.9%), sclerotherapy (58.6%), surgery (42.5%), close follow-up (47.1%), none (0%) (Figure 7).

Moreover, 97% of participants considered it possible to intervene with appropriate therapies to delay or counteract the formation of corona phlebectatica; in particular, this includes medical/pharmacological therapy [GAGs, sulodexide, micronized purified flavonoid fraction (MPFF)] (77.0%), elastocompressive therapy (72.4%), lifestyle (17.2%), reflux correction, insole, and compliance (5.7%), and surgical therapy (4.5%) [21]. It should be noted that this is an evaluation for re-operation to correct the haemodynamic disorder, as in the previous answer, almost two thirds of the respondents consider haemodynamic correction to be essential.

In the presence of VLUs, 77.0% of specialists consider drug therapy [21] [glycosaminoglycans (GAGs), sulodexide, micronized purified flavonoid fraction (MPFF)] in combination with compression therapy (72.4%).

About 90.8% consider that “wound care” has a relevant impact to prevent venous ulcer recurrence. In relation to the prevention of recurrence of ulcers, the respondents consider the following as very important: accurate hemodynamic evaluation (97.7%), re-evaluation of compression and pharmacological therapy (94.3%), correction of reflux (92.0%), treatment of PTS (85.5%), and assessment of patient compliance and comorbidity (87.4%).

## 4. Discussion

This survey was submitted to vascular specialists, the vast majority of whom were vascular surgeons and angiologists. The responding physicians were divided equally into the two geographical macro-areas of professional affiliation: North and Centre–South Italy. The northern macro-region was more represented in the general sample.

The management of CVD may be different in some geographical areas of Italy due to different healthcare regional organizational settings. The majority of physicians demonstrated good professional experience (between 10 and 20 years of activity) and worked in mostly public hospital structures. Most of the respondents stated that they carry out vascular diagnostics and manage the venous pathology overall (diagnosis, treatment, follow-up) in their centre.

According to the data of the survey, about half of the vascular specialists involved find the presence of “corona phlebectatica” in their patients in a percentage that ranges between 20% and 50% of cases. The majority of attendees (73.6%) consider the presence of the “corona phlebectatica” as a moderate/high unfavourable prognostic factor for the evolution of the disease. White atrophy, PTS, and hypodermitis were considered other risk factors for evolution. Data from the responses of the survey are substantially in agreement with the data in the literature. Indeed, a recent systematic review on CVD shows an extremely relevant prevalence of the various CEAP classes.

Considering the classes C2–C5, we would have a total of about 30–35% of the population (C2: 19%; C3: 8%; C4: 4%; C5: 1%) [11]. The clinical relevance of the “corona phlebectatica” in our survey agrees with the considerations of some studies in the literature. A study of nearly 900 patients, evaluated by 49 specialists, demonstrated a statistical association of “corona phlebectatica” with more advanced forms of CVD in class C [22]. Other studies demonstrated good sensitivity (but low specificity) of “corona phlebectatica” with the more advanced forms of class C [23]. Indeed, patients with “corona phlebectatica” display a 5.3 times greater risk of developing an ulcer [9].

Half of the respondents found a venous ulcer recurrence in over 15% of patients, and the recurrence occurs between 1 and 5 years for almost all (96%) attendees. This answer confirms data that can be found in the literature, albeit with some differences. Indeed, some studies showed that the recurrence of a healed ulcer can occur in over 70% of patients three months after healing [18,19]. The data from the questionnaire relating to over 15% of relapses may represent a broad spectrum that is difficult to compare and therefore partially imprecise, due to the type of data collection through a questionnaire, but clearly indicates a trend of general agreement with the data in the literature.

Most of the attendees consider PTS to be a high-risk factor for venous leg ulcer recurrence. Poor compliance of the patient is considered the major risk factor for recurrence of VLUs by almost all respondents.

Lack of use of elastic compression, obesity, and lack of medical therapy are other major risk factors. In the event of venous ulcer recurrence, the most relevant behaviours that are observed by respondents, in order of relevance, are the hemodynamic re-evaluation by duplex ultrasound, re-evaluation of compression and medical therapy, treatment of the hemodynamic disorder, treatment of PTS, control patient compliance, and comorbidity.

Almost all of the specialists participating in the survey argued that it is possible to contrast/slow down the progression of CVD in the presence of “corona phlebectatica” as well as to prevent the recurrence of venous ulcers. The aims of VLU treatment are to promote wound healing, reduce pain and oedema, improve the quality of the patient’s life, and prevent ulcer recurrence.

Systemic treatment includes weight reduction among patients with obesity, and routine physical activity. Localized specific treatments include compression stockings and/or surgical procedures such as sclerotherapy and topical laser therapy [24].

The vast majority of participants consider elastic compression therapy (77%) and pharmacological therapy (72.4%) as essential, in particular GAGs, sulodexide, and MPFF. The re-evaluation/further correction of reflux, weight loss, skin care, physical activity, infection prevention, patient compliance, and hydration are considered as relevant behaviours. Those data are in agreement with the results of a recent Cochrane systematic review evaluating compression bandages or hosiery for preventing the recurrence of venous ulcers [25]. Compression with European class 3 compression stockings can decrease reulceration compared with no compression over 6 months [25]. Use of European class 1 compression stockings compared with European class 2 compression stockings cam result in little or no difference in reulceration and noncompliance over 12 months [25]. UK class 3 compression hosiery can decrease reulceration compared with UK class 2 compression hosiery; however, higher compression may lead to lower compliance [25]. There may be little to no difference between Scholl and Medi UK class 2 compression stockings in terms of reulceration and noncompliance [25]. Compression therapy is based on the concept of applying external pressure on the limb to improve venous hemodynamics, to control oedema, to reduce inflammatory mediators, to improve microcirculation, to improve arterial inflow, and to improve lymphatic drainage.

It is relevant to perform a patient’s overall assessment for the attendees of this survey, risk factors, comorbidities, and compliance, and hence the need for closer clinical control. Furthermore, the need for hemodynamic evaluation/re-evaluation and its possible correction together with compression therapy in association with drug therapy (GAGs, sulodexide, MPFF) emerges as a cornerstone therapy.

Most patients with VLUs will be followed by a primary care physician, who may delegate some basic diagnostic procedures to services of community nurses or nurses specializing in wound care. After the initial appearance of an ulcer, the primary care physician can refer the patient to a specialist for a further assessment. This is usually a vascular surgeon, an angiologist, or a dermatologist for a vascular evaluation, or in case of a strong delay in healing, a dermatologist in order to exclude other differential diagnoses.

The treatment (compression treatment, drugs, or local care of the wound) is prescribed by the general practitioner or one of the specialists consulted, and it can be carried out by paramedical staff, community nurses, or specialist nurses in wound care at specialist treatment centres. Moreover, if patients are increasingly treated in outpatient settings, in most countries, due to the growing need to reduce healthcare costs, patients can still be admitted to a hospital for their treatment if they have difficult injury recovery or need to undergo surgery. Networks should be established between hospitals and nursing services’ community or wound care centres to ensure continuity of the care of patients after their discharge from a hospital.

### Study Limitations

The sample of specialists involved in the survey satisfies a good part of the requisites that can be considered reliable for the reliability of the questionnaire. Indeed, they were vascular specialists with extensive professional experience. The greater representation of specialists in the macro-geographical area of northern Italy could make the data less reliable at a national level, although considering only two macro-geographical areas (North and Centre–South), the territorial subdivision would be substantially balanced. However, it is evident that the specialists involved, although very reliable, cannot be considered representative of the general picture. Furthermore, in some multiple-choice questions, it was not possible, due to the setting of the questionnaire itself, to separate some data in a more analytical way. The study analyses the experience of vascular specialists in treating this condition, reporting their own personal experience (self-reporting bias). This study does not evaluate certain patient-related outcomes.

## 5. Conclusions

Data of our survey lead to some considerations useful for clinical practice in the management of CVD in its various phases:

(a) High frequency of venous ulcer recurrence at over 15%.
(b) Short times for the development of venous ulcer recurrence (1–5 years).
(c) The presence of “corona phlebectatica” is an unfavourable prognostic marker towards the progression of CVD.
(d) Importance of the analysis of patient-related risk factors and strict re-evaluation of the patient.
(e) The extreme importance of a combined approach of compression therapy + pharmacological therapy (GAGs, sulodexide, MPFF), contextually (before, waiting, after) of any hemodynamic correction.
(f) Networks should be established between hospitals and primary care physician and nursing services’ community or wound care centres to ensure continuity of the care of patients.

### Key Messages Section

The presence of “corona phlebectatica” is an unfavourable prognostic marker towards the progression of CVD.

It is relevant to perform an analysis of patient-related risk factors and a strict re-evaluation of the patient.

A combined approach of compression therapy + pharmacological therapy (GAGs, sulodexide, MPFF), contextually (before, waiting, after), is the most relevant to any hemodynamic correction.

## Data Availability

Data are available on request.
